# How POCUS picks up a rare mimickers of acute appendicitis in the emergency department

**DOI:** 10.1186/s13089-020-0151-6

**Published:** 2020-01-15

**Authors:** Shihab Al Sheikh, Mariam Al Ali, Dhanya Sochel Kiran, Mohsen Al Tabatabai

**Affiliations:** Emergency Department, Rashid Hospital Trauma Center, Dubai, United Arab Emirates

**Keywords:** POCUS, Point of care ultrasound, Acute appendicitis mimicker, Computed tomography, Emergency department

## Abstract

**Background:**

Appendicitis is the most common surgical abdominal emergency. Punctual diagnosis and rapid operative treatment of acute appendicitis are critically important, as it reduces the risk of complications, associated with greater morbidity and cost of care. The clinical diagnosis of acute appendicitis can be difficult and confusing. Due to some typical presentation and mimic, several abdominal conditions are left undiagnosed. POCUS has comparatively acceptable sensitivity and high specificity for diagnosing acute appendicitis, and early practice POCUS has a standalone test to rule out acute appendicitis.

**Case presentation:**

A 43-year-old man presented with a 3-day history of abdominal pain rated 5/10 in intensity which had localized to the right iliac fossa by the time he attended our emergency. He described the acute pain as sharp in nature, colicky without the presence of any radiation. The pain was associated with nausea, but there was no vomiting. On clinical examination, the patient was stable at first, had a normal temperature with tenderness and guarding the right iliac fossa under nearby normal laboratory investigation.

**Conclusion:**

The importance of POCUS in scanning right iliac fossa for patients present with signs and symptoms that are mimicking acute appendicitis for diagnosing a rare pathology and avoiding the risk of ionizing radiation hazards and unnecessary surgical intervention.

## Background

Appendicitis is the most common surgical abdominal emergency worldwide with more than 250,000 people diagnosed annually, and 7% of the population having the disease in their lifetimes [1]. A life table model suggests that the lifetime risk of appendicitis is 8.6% for males and 6.7% for females; the lifetime risk of appendectomy is 12.0% for males and 23.1% for females [2]. Punctual diagnosis and rapid operative treatment of acute appendicitis are critically important, as it helps in the reduction of the risk in case of certain complications, associated with greater morbidity and cost of care [3, 4]. The clinical diagnosis of acute appendicitis can be difficult and confusing due to an atypical presentation and mimic several abdominal conditions [5]. Patients with many other disorders have symptoms similar to those of appendicitis, such as acute gastrointestinal diseases like Crohn’s disease, infectious enterocolitis, mesenteric adenitis, cecal diverticulitis, Meckel’s diverticulitis, epiploic appendagitis, and omental infarcts can present with right lower quadrant. In addition, acute genitourinary diseases, like pyelonephritis and ureterolithiasis, also have similar symptoms. In a young woman, acute gynecological disease processes, such as ovarian torsion, hemorrhagic ovarian cyst, pelvic inflammatory disease, and ectopic pregnancy, should also be considered within the differential diagnosis [6].

In 1981, Preusser published a first case report in an 87-year-old man, with typical symptoms and clinical signs of acute appendicitis where he was able to demonstrate the swollen and fluid-filled appendix by sonography that was confirmed at operation [7]. Pyualert et al. 5 years later described the ‘graded compression technique’ for the sonographic examination of the appendix with high-frequency transducer [8]. The POCUS for the diagnosis of appendicitis has reported sensitivities of 75% to 90%, specificities of 83% to 95%, positive LR of 4.5 to 5.8, negative LRs of 0.19 to 0.27, and positive predictive value of 90% [9].

## Case presentation

A 43-year-old man presented with a 3-day history of abdominal pain rated 5/10 in intensity which occurred at the right iliac fossa by the time he attended our emergency. He described the acute pain, colicky, and there was no radiation. The pain was associated with nausea, but no vomiting occurred. He had no bowel or urinary symptoms and no previous abdominal problems. He had no significant past medical and family history.

On examination: vital signs were temperature: 36.8 °C, pulse: 80 per min, blood pressure: 116/78, SPo2: 99%. Abdominal examination findings revealed a soft abdomen with tenderness and guarding in the right iliac fossa. There was no rebound tenderness. Rovsing’s, psoas and obturator signs were negative.

## Investigation

Laboratory investigations revealed his peripheral white blood cell count to be 9.900/µL and the neutrophil level was 7.400/µL (3.9–11.0/µL). His serum C-reactive protein level was 0.6 mg/L (< 0.5 mg/L), and serum creatinine was 1.2 mg/dL (0.7–1.2 mg/dL). Urine analysis has revealed that blood by strips 5 + , RBC/HPF 10–15, WBC/HPF 0–s5 and epithelial cells are few in number.

**Fig. 1:**
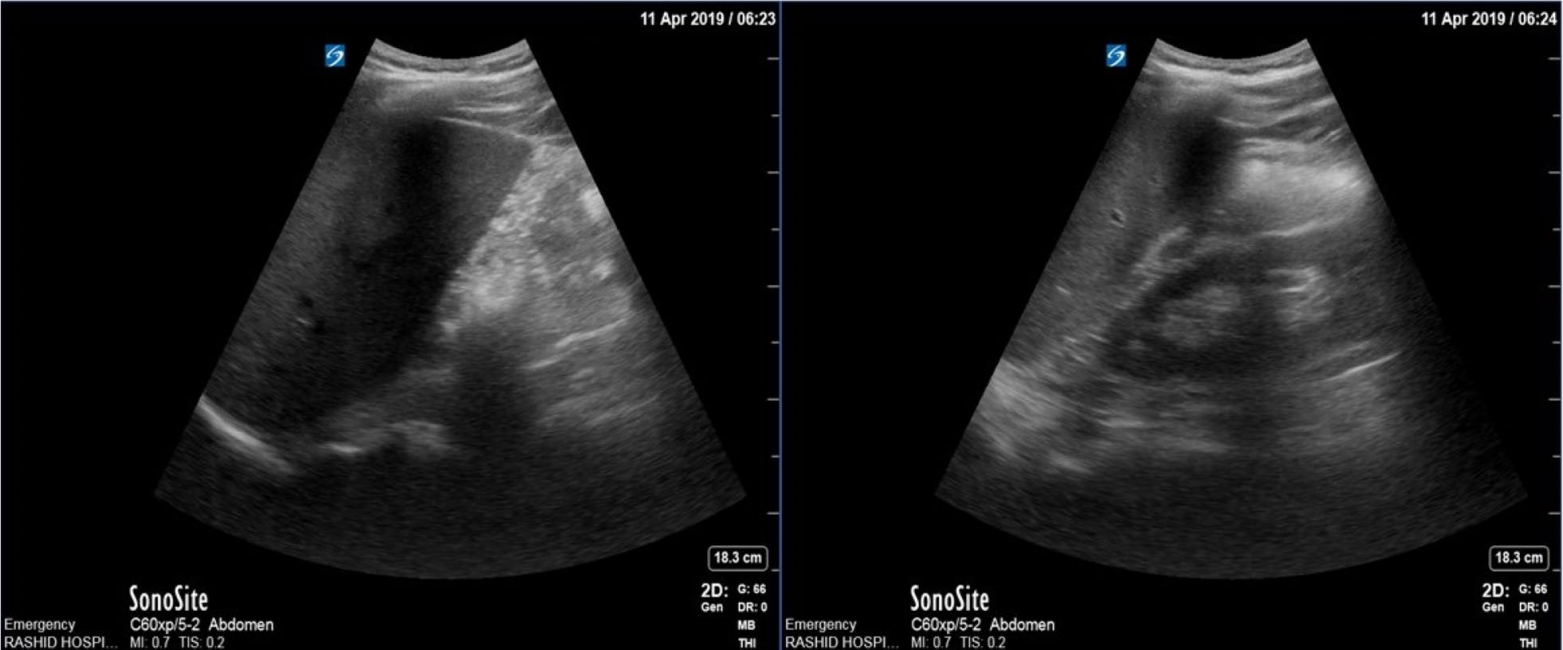
Left side showing the absence of the right kidney where the left kidney in the normal position

**Fig. 2:**
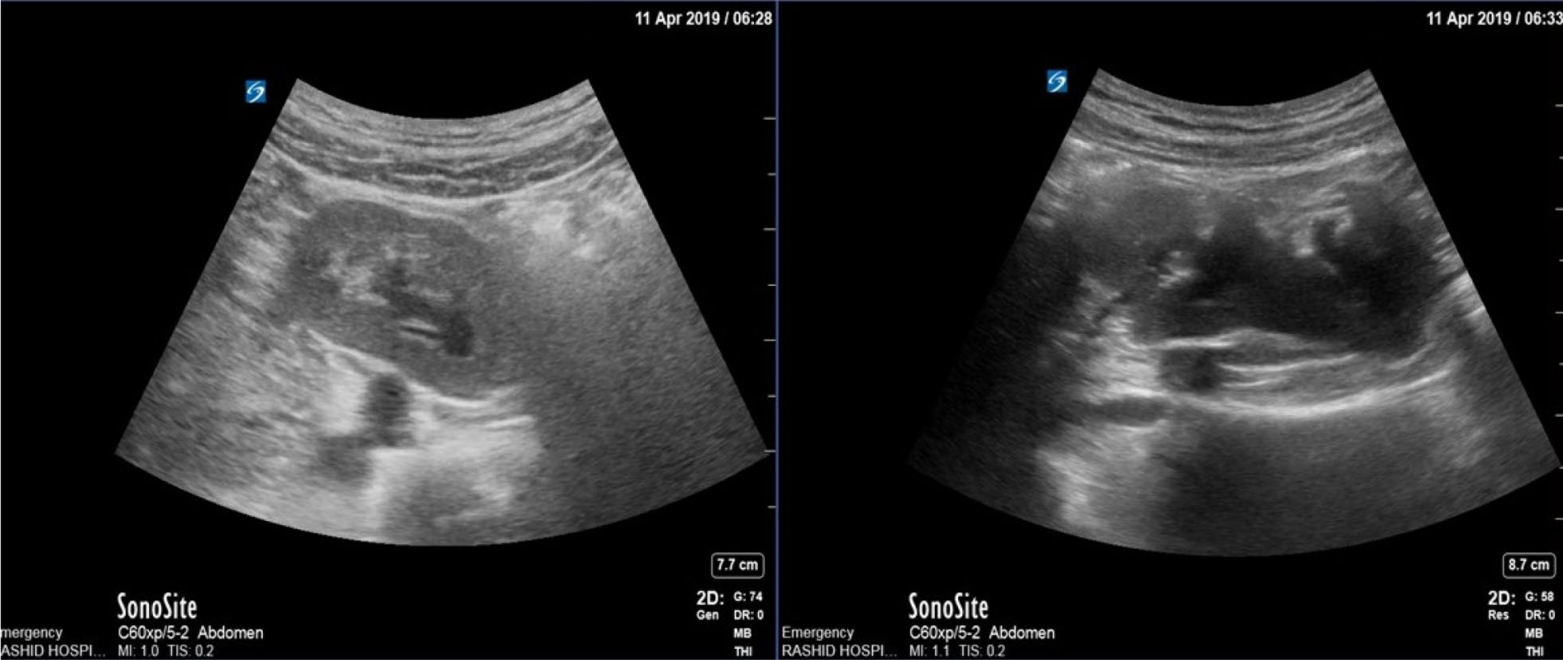
Left side showing the transverse section through the right ectopic kidney and right side through the longitudinal section

**Fig. 3:**
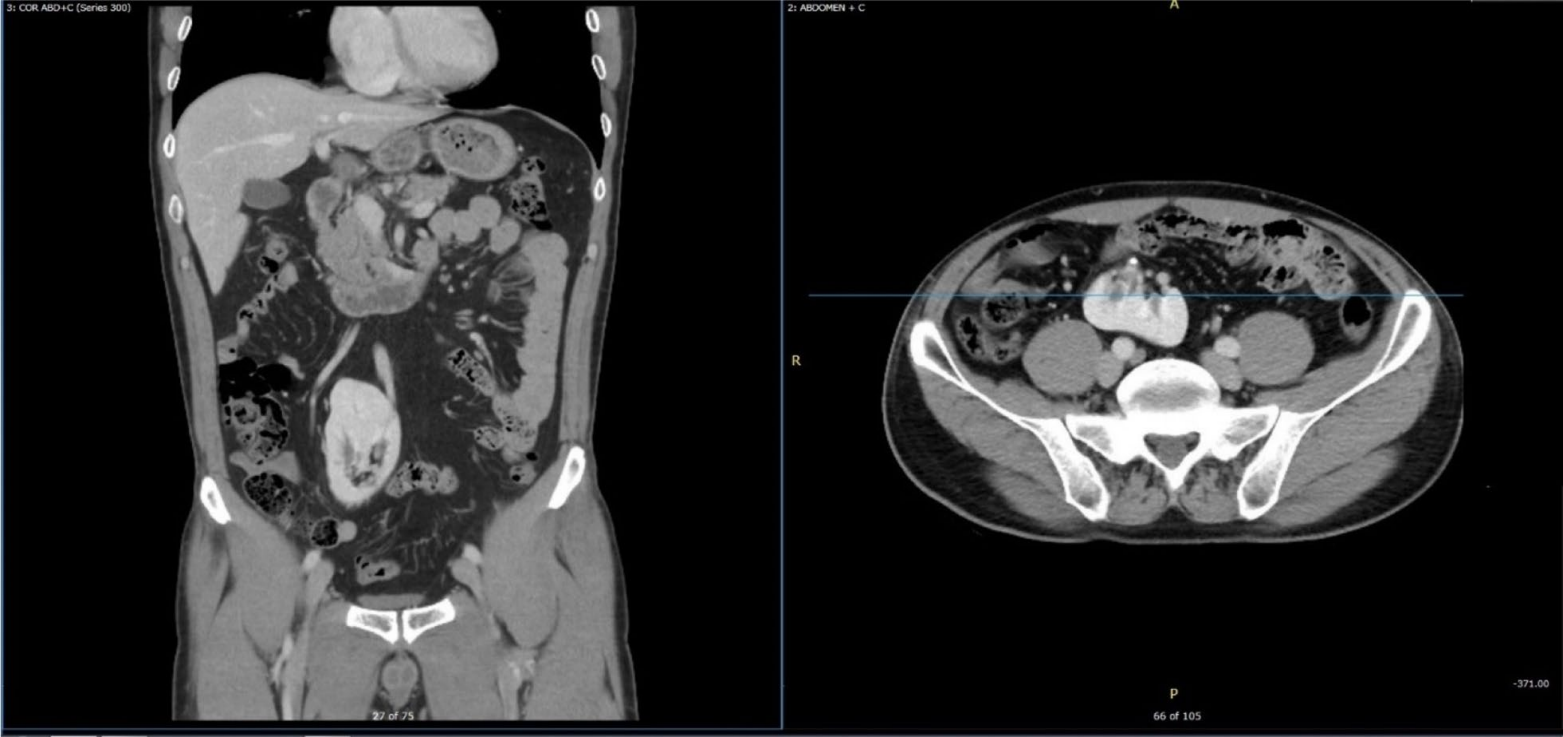
CT abdomen, showing anterior malrotated ectopic right kidney with small stone at pelviureteric junction

According to the physical examination and lab tests, acute appendicitis was the most likely form of diagnosis. POCUS was done using a curvilinear probe and started in the right lower quadrant at the point of maximum tenderness. This was mainly carried out to explore the inflamed appendix, where we found a kidney-shaped mass at the right iliac fossa. An appendix could not be visualized in a clear manner. Thus, we scanned both flanks and found that the right kidney was absent, while the location of the left kidney was normal. This alarmed us of the ectopic right pelvic kidney **(**Fig. 1). On detailed evaluation, we identified the mass as a kidney with mild hydronephrosis **(**Fig. 2). After the pain was managed with adequate analgesics, we proceeded to do a CT with contrast. This revealed a normal left kidney and right ectopic kidney which had 2 small stones of size 3 mm and 6 mm at its renal pelvis and pelviureteric junction. It was also observed that the left kidney was affected by secondary mild hydronephrosis, where the appendix is normal **(**Fig. 3).

## Discussion

The first observation on the solitary ectopic pelvic kidney was made by Henot in 1830. It was made on an autopsy of 8 months of age whose sex was indefinite owing to the absence of the genitalia [10]. Necropsy records were derived from many sources which had indicated that ectopic kidney is found once in from 2150 to 3000 [11]. There are many factors that inhibit the kidney from gradual ascend to the abdomen and away from the midline. The factors can be in the form of ureteral bud maldevelopment, defective metanephric tissue, genetic abnormalities, maternal illness and teratogenic causes [12]. Clinically the renal ectopia is more readily recognized in females because they undergo uro-radiological evaluation more frequently than that of males. As a result of which, a higher rate of urinary tract infection gets associated with genital anomalies [13]. Ectopic kidneys are usually smaller than the normal size of kidneys. The renal pelvis is usually anterior to the parenchyma because it is incompletely rotated. It has been observed that the major portion of the ectopic kidneys is clinically asymptomatic. Ectopic kidneys are no more susceptible to disease than normal. Exceptions are there such as hydronephrosis development, renal stones formation, and urinary tract infections [14].

**Fig. 4:**
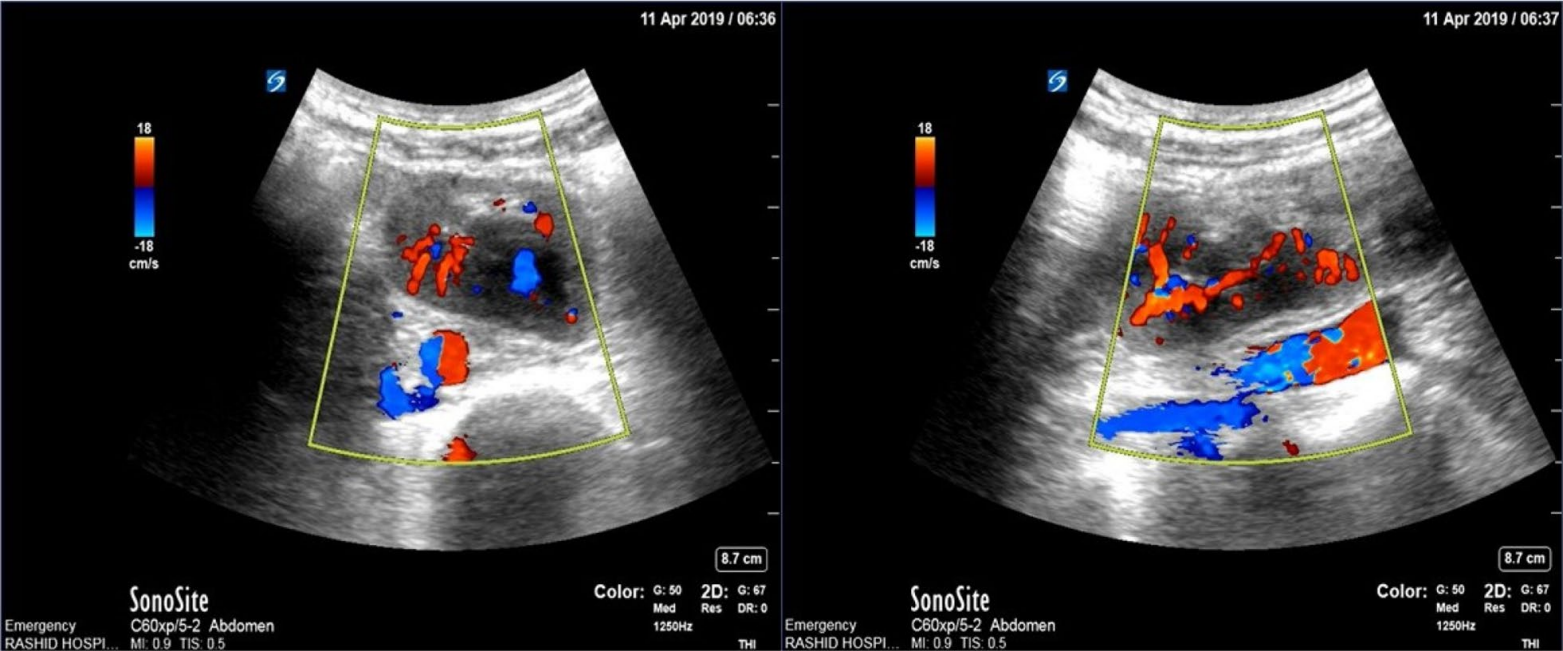
The color Doppler of great value by demonstrating a normal vascular architecture that is compatible with renal vessels

In our case, the unnecessary surgical operation was avoided by just 2 min. With the implementation of POCUS and diagnosis, it was confirmed that CT abdomen with IV contrast. Ultrasound can recognize the ectopic kidney by its overall similarity in shape, size and structure to normal kidneys. However, the pelvic kidneys might get developed with unusual shapes and degrees of rotation can form. Moreover, it may show some dilatation of the collecting system [15]. These unusual features may make an ectopic kidney difficult to recognize as a kidney, especially if an unexpected finding is observed which might present a mass during an examination. This can be highly confusing, as it can have similarities with appendicular mass, bowel tumors, and pelvic lymphadenopathy. Here the color Doppler is of great value as it demonstrates a normal vascular architecture that is compatible with renal vessels [16] (Fig. 4).

Both POCUS and abdominal helical computed tomography (CT) are essential tools in managing the patient who is affected with acute abdominal pain in the emergency department. Both are considered to have acceptable sensitivities, specificities, and positive and negative predictive values. It has been regarded that CT to be more superior in numerous studies [17]. Although with the advantage of the high sensitivity of abdominal CT, there are mainly three disadvantages of the abdominal CT. The first is exposing the patient to the risk of ionizing radiation, with an estimated 2% of future cancers being triggered just by CT scans [18]. The second disadvantage is that the CT abdomen is highly expensive and is not accessible in all medical providing institutions primarily in developing countries. Finally, prolonged emergency department stays when CT abdomen order with oral and/or rectal administration. It has also been inculcated that there is a risk of allergic reaction and nephrotoxicity from IV contrast administration. The advantages of the POCUS are lack of ionizing radiation, noninvasive, simple to handle, document the entire ultrasound finding, widely available, real-time imaging, portable and cheap. Furthermore, repetitive ultrasound examinations can be done easily simultaneously which leads to enhancing clinicians’ ability to perform serial reassessments and assists in further management. POCUS is recognized to be useful in children and pregnant patients and is one of the principal modality for these patients based on the American College of Radiology guidelines [19]. The disadvantages are decrease sensitivity, lack of operator experience, patient factors like obesity. A few of the other disadvantages can be superimposed bowel gas or typically located appendix. There can also be greater pain during the application of the graded compression process. Poortman et al. [20] concluded that a diagnostic pathway includes the initial US and complimentary CT in patients with negative or inconclusive US. The results yield high diagnostic accuracy in the management of acute appendicitis without adverse events.

## Conclusion

This case report highlights the importance of POCUS for scanning the right iliac fossa for patients with suspicion of acute appendicitis before any hazardous ionizing radiation imaging or surgical intervention. Thus, it can be considered that the calculus of renal pain of an ectopic right pelvic kidney in the differential diagnosis falls under the list of right iliac fossa pain.

## Data Availability

Not applicable.

## Abbreviations

POCUS: point-of-care ultrasound
CT: computed tomography
IV: intravenous
